# Levamisole tainted cocaine causing severe neutropenia in Alberta and British Columbia

**DOI:** 10.1186/1477-7517-6-30

**Published:** 2009-11-17

**Authors:** Lewinda Knowles, Jane A Buxton, Nataliya Skuridina, Ifeoma Achebe, Donald LeGatt, Shihe Fan, Nancy Yan Zhu, James Talbot

**Affiliations:** 1 Edmonton Zone Medical Office of Health, Alberta Health Services, Suite 101 West Tower, 14310-111 Avenue, Edmonton, AB (T5M3Z7), Canada; 2 Epidemiology Services, British Columbia Centre for Disease Control 655 West 12th Ave, Vancouver British Columbia (V5Z 4R4), Canada; 3 School of Population and Public Health, University of British Columbia, 5804 Fairview Avenue, Vancouver British Columbia, (V6T 1Z3), Canada; 4 Department of Medicine (Community Medicine), University of Alberta, Suite 4000 RTF, 8308 - 114 Street, Edmonton, Alberta (T6G 2V2), Canada; 5 Department of Laboratory Medicine & Pathology, 4B4.08 Mackenzie Health Sciences Centre, University of Alberta Hospitals, Edmonton, Alberta (T6G 2R7), Canada; 6 Department of Medicine (Hematology & Clinical Oncology), University of Alberta, 2E3 Walter Mackenzie Centre, Edmonton, Alberta (T6G 2B7), Canada; 7 Department of Public Health Sciences, University of Alberta, 3-50 University Terrace, 8303 - 112 Street, Edmonton, Alberta (T6G 2T4), Canada

## Abstract

**Background:**

Five cases of severe neutropenia (neutrophil counts < 0.5 per 10^9 ^cells/L) associated with exposure to cocaine and levamisole, an antihelimithic agent no longer available in Canada, were identified in Alberta in 2008. Alberta and British Columbia (BC) public health officials issued an advisory and urged health care professionals to report cases to public health. This paper presents the findings of the public health investigations.

**Methods:**

Cases were identified prospectively through reporting by clinicians and a retrospective review of laboratory and medical examiners data from January 1, 2006 to March 31, 2009. Cases were categorized as confirmed, probable or suspect. Only the confirmed and probable cases are included in this paper.

**Results:**

We compare cases of severe neutropenia associated with tainted cocaine (NATC) identified in Alberta and BC between January 1, 2008 to March 31, 2009. Of the 42 NATC cases: 23(55%) were from Alberta; 19(45%) were from British Columbia; 57% of these cases reported crack cocaine use (93% of those who identified type of cocaine used); 7% reported using cocaine powder; and the main route of cocaine administration was from smoking (72%). Fifty percent of the NATC cases had multiple episodes of neutropenia associated with cocaine use. Cases typically presented with bacterial/fungal infections and fever. One Alberta NATC case produced anti-neutrophil antibodies, and four were positive for anti-neutrophil cytoplasmic antibody (ANCA). Analysis of two crack pipes and one drug sample obtained from NATC cases confirmed the presence of both cocaine and levamisole. A further 18 cases were identified through the retrospective review of laboratory and medical examiner data in Alberta

**Interpretation:**

Our findings support a link between neutropenia and levamisole tainted cocaine; particularly from smoking the crack form of cocaine. Some patients may be genetically predisposed to develop levamisole-related neutropenia. Awareness of the differential diagnosis will assist clinicians with case timely detection and appropriate management.

## Introduction

The modification of illicit drugs is not an uncommon phenomenon. In efforts to enhance the profitability and acceptability of a product, illicit drugs typically undergo processes such as: substitution (replacement of one drug for another with similar pharmacologic properties); dilution (addition of inert substance to reduce the content of the active drug); contamination (unintentional inclusion of a foreign substance); and/or adulteration (intentional addition of a substance with: i)similar pharmacologic properties or ii)properties which attenuate the effects of the parent drug)[1]. Adverse health effects from modified cocaine are varied and have been previously reported in Scotland [2], Britain [3], Switzerland [4], and Philadelphia, USA [5].

Since 2004, the emergence of a cocaine modifier called levamisole has been reported in Canada [6], United States [7-9], United Kingdom [10] and Italy [11]. The use of levamisole, an antihelmithic agent and cancer drug, was discontinued in Canada in August 2005. However, levamisole is still used for veterinary medicine in the United States and South America. It is estimated that 11% of cocaine samples seized in Alberta, Canada test positive for levamisole (April to December 2008)[12]; and 47% of samples tested in the United States [13]. The reason levamisole is being added to cocaine is unclear.

In 2008-2009, both Alberta and British Columbia public health officials investigated clusters of severe neutropenia associated with levamisole modified cocaine use; particularly in association with the smoking of crack cocaine. We present the findings from our investigations to increase awareness in clinicians and to improve the identification of cases.

## Methods

In 2008, clinicians notified public health officials of five cases of severe neutropenia in Northern Alberta; cocaine and levamisole were detected in the urine of all five cases [14]. On November 21, 2008, Alberta Health Services disseminated a public health advisory to community partners and healthcare professionals [15]. The advisory highlighted the link between agranulocytosis and cocaine tainted with levamisole, the process for submitting urine samples for cocaine and levamisole toxicology, how to report cases and recommendations regarding case management. A broader provincial and national advisory shortly followed this communication. In response to Alberta's advisory and the identification of similar cases, the British Columbia Ministry of Health issued a provincial advisory on December 11, 2008 [16].

On November 18, 2008, the Clinical Toxicology Laboratories at the University of Alberta Hospital and *Dyna****LIFE***_*DX *_in Edmonton began to append a clinical alert on all laboratory reports testing positive for cocaine. This alert highlighted the relationship between neutropenia and cocaine tainted with levamisole. Identification of levamisole in urine was limited to a few facilities in Alberta and none in British Columbia. The University of Alberta Hospital Toxicology Laboratory in Edmonton agreed to conduct levamisole testing on behalf of British Columbia. A literature review was performed to inform the investigation.

### Study Design

This investigation focused on observational prospective and retrospective case reports of neutropenic patients associated with cocaine use in Alberta and British Columbia between January 1, 2006 and March 31, 2009.

### Data collection and abstraction

Patients presenting with severe neutropenia (defined as neutrophil counts less than 0.5 per 10^9 ^cells/L), and recent cocaine use in Alberta or British Columbia between January 1, 2006 and March 31, 2009 were identified as cases of Neutropenia Associated with Tainted Cocaine ("NATC"); specifically, levamisole tainted cocaine. Cases were categorized as confirmed, probable, or suspect NATC cases (see Appendix 1). Only confirmed and probable NATC cases are presented in this paper.

Prospective NATC case identification relied on clinical professionals to identify and report patients who met NATC case definitions to public health, who followed up to obtain additional information. Alberta collected common data elements from attending physicians, medical records and interviewed the NATC case, when possible. British Columbia developed a standardized data collection form for clinicians to report NATC cases to public health. NATC cases were excluded when medical evidence supported an alternative justification for neutropenia (e.g. chemotherapy).

Alberta performed retrospective chart review using laboratory and medical examiner data. Retrospective laboratory data was obtained from the Edmonton, Calgary, Chinook, East Central and Peace areas of Alberta between January 1, 2006 and March 31, 2009. NATC cases identified through the laboratory and medical examiner data review processes involved searching for potential cases with concurrent laboratory results indicative of severe neutropenia and positive cocaine, cocaine metabolites and/or levamisole screens. Where possible these NATC cases were further cross-referenced with electronic medical records, to determine any NATC exclusion factors and documented risk factors.

Health Canada Drug Analysis Service provided testing for cocaine and levamisole markers in suspected cocaine samples and paraphernalia. Toxicology Laboratories in Edmonton and Calgary tested urine for cocaine, its metabolites, and levamisole; the University of Alberta Hospital Toxicology Laboratory also tested drug paraphernalia related to current patients. Clinicians were requested to collect urine specimens for toxicology testing from identified neutropenic patients if within 48 hours of cocaine consumption. Typically, neutrophil counts were performed when patients sought medical care.

## Results

**Figure 1:**
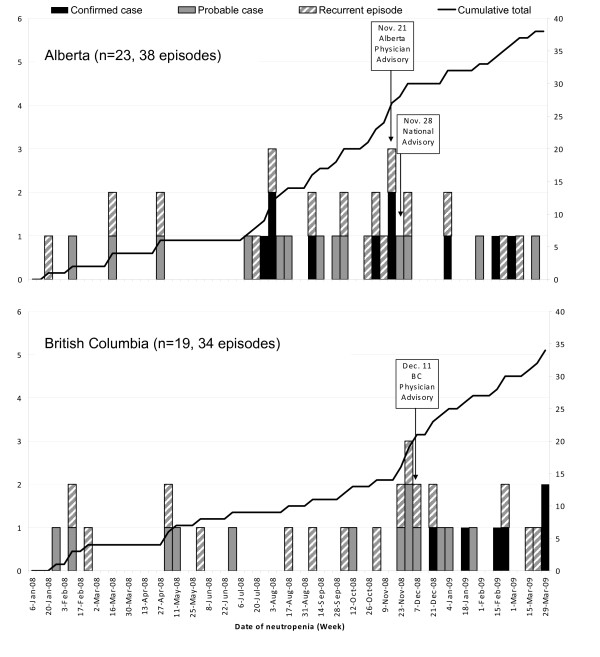
Neutropenia associated with levamisole tainted cocaine episodes cocaine use in Alberta (A) and British Columbia (B), Canada, 2008-2009

Forty-two cases of NATC were identified in Alberta and British Columbia from January 1, 2008 to March 31, 2009. In this time period, 16 confirmed, and 26 probable, NATC cases were identified. Eighteen (43%) NATC cases had recurrent episodes of neutropenia associated with cocaine use (range: 2 to 8 episodes). The dates of NATC case identification are shown in Figure 1. Characteristics of these 42 NATC cases are presented in Table 1; 64% of cases were female. Of the NATC cases where cocaine details were obtained, most (93%) used crack cocaine; two probable cases reported only using cocaine powder. The main route of cocaine consumption was smoking (72% where route was known).

**Table 1:** Characteristics of neutropenia associated with levamisole tainted cocaine (NATC) cases in Alberta and British Columbia, January 2008 -- March 2009

*Characteristic:*	*Alberta*		*British Columbia*		*Total*	
No. of NATC cases	23		19		42	
*Confirmed (%)*	*10*	*(43)*	*6*	*(32)*	*16*	*(38)*
*Probable (%)*	*13*	*(57)*	*13*	*(68)*	*26*	*(62)*
No. of NATC episodes	43		45		88	
Mean age, years (range, years)	39	(18-52)	36	(22-63)	37	(18-63)
Gender						
Males *(%)*	9	(39)	6	(32)	15	(36)
Females *(%)*	14	(61)	13	(68)	27	(64)
Type of cocaine exposure						
Crack *(%)*	13	(57)	11	(58)	24	(57)
Powder *(%)*	0	(0)	2	(11)	2	(5)
Both *(%)*	0	(0)	1	(5)	1	(2)
Unknown	10	(43)	5	(26)	15	(36)
Route of cocaine exposure**						
Smoke *(%)*	8	(35)	10	(53)	18	(43)
Snort *(%)*	0	(0)	7	(37)	7	(17)
Inject *(%)*	0	(0)	1	(5)	1	(2)
UNKNOWN *(%)*	15	(65)	2	(11)	17	(40)
No. of NATC cases with repeated neutropenia episodes (range, No. of episodes)	8	(2-7)	10	(2-8)	18	(2-8)
No. of NATC cases that had bone marrow biopsies (%)	8	(35)	7	(37)	15	(36)

* Testing information was not reported for all cases.

** Some NATC cases reporting using cocaine by more than one method. As such, the sum of the percentages will not equal 100.

Bacterial and fungal infections reported in patients with neutropenia included: abscesses, bacteremia, cellulitis, urinary tract infection, pneumonia, invasive group A streptococcus, septic shock, epiglottitis, ulcers(peptic, skin, esophageal), and thrush. Of the 15 NATC cases who underwent bone marrow biopsy assessment, 12 (80%) cases had the procedures prior to distribution of the public health advisories.

Reported history of cocaine use varied from occasional use to chronic use and binging. Ten of the 16 confirmed NATC cases (63%) used crack cocaine within two days of seeking medical care, some within hours of seeing a physician. Five NATC cases indicated heavy crack cocaine usage (1 to 3 grams per day) just prior to admission.

**Figure 2:**
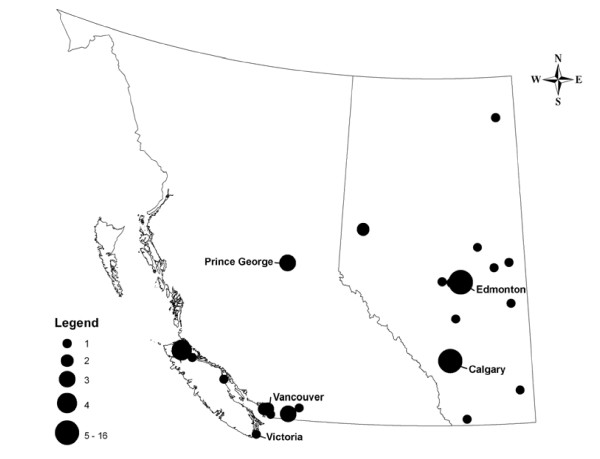
Distribution of neutropenia cases associated with cocaine use in Alberta and British Columbia (n = 60), Canada, 2006-2009

NATC cases resided in both large urban centres and rural communities (see Figure 2). In British Columbia most cases occurred in rural communities.

Some differences in NATC case characteristics between the Alberta and British Columbia cohorts were noted. British Columbia identified 13 (68%) NATC cases of aboriginal heritage, four cases (17%) in Alberta were identified as Aboriginal. In Alberta, one death was associated with the consumption of levamisole tainted crack cocaine. One NATC case in Alberta was tested for and produced anti-neutrophil antibodies, both IgG and IgM subtypes, as detected by flow cytometry and HLA Class I antigens. For another five NATC cases, anti-neutrophil cytoplasmic antibody (ANCA) tests were conducted; four NATC cases were positive (two for pANCA; two for cANCA).

The contents of two used crack pipes obtained from NATC cases verified the presence of cocaine and levamisole. One sample of cocaine was tested for levamisole and found to be positive; quantifying the percentage of levamisole in the sample was not possible in Canada at that time.

A further 18 cases (20 episodes) were identified through the retrospective review of laboratory and medical examiner data in Alberta between January 1^st ^2006 to December 31^st ^2007. The earliest confirmed NATC case dated back to July 2007 and the earliest probable NATC case dated back to June 2006.

## Discussion

We identified a total of 60 NATC cases and 108 episodes of neutropenia associated with levamisole-tainted cocaine, in Alberta and British Columbia since June, 2006. Most cases were related to smoking crack, and some cases reported heavy use prior to seeking medical care; though we were unable to confirm a dose response.

Literature suggests that levamisole remains stable when heated [17], but may potentiate the nicotinic acetylcholine receptors of the human central nervous system and act as a ganglion nicotinic acetylcholine receptor agonist [18,19]. Levamisole has also been found to increase dopamine and endogenous opiate (morphine, codeine) levels in the brains of rats [20]. However, it remains unknown where levamisole is added to the cocaine and for what purpose.

Some patients may be genetically predisposed to develop levamisole-related neutropenia. Prior studies found people with levamisole-related neutropenia were more likely to have HLA-B27, an HLA class I antigen [21]. As routine HLA-B27 testing is difficult, the utility of this risk factor is unknown.

Levamisole is known to have immunostimulating effects with the production of auto-antibodies [22]. Anti-neutrophil antibodies found in patients who develop neutropenia after levamisole use have been postulated as a potential cause for the neutropenia [23]. ANCA have also been implicated in drug-induced neutropenia [24]. In our investigation we found one case positive for anti-neutrophil antibodies and four positive for ANCA, which support the speculation that these auto-antibodies may cause levamisole-related neutropenia.

Despite public health notification and media interest in both provinces the true burden of NATC is likely underestimated by voluntary reporting of NATC cases by clinicians. As levamisole has a short half-life (approximately 5 to 6 hours) and little (2 to 5%) is excreted unchanged in urine, specimens should be collected within 48 hours of exposure [25,26]. Thus delayed identification of NATC cases may have led to missed urine levamisole testing and case confirmation. The misclassification of NATC cases based on other competing health conditions may have occurred. Finally, NATC case findings were limited by the lack of accessibility to retrospective laboratory data and the availability of levamisole testing in British Columbia.

Clinicians should be aware that severe neutropenia may be caused by levamisole in cocaine. If fever or infection is present, empiric intravenous broad spectrum antibiotics and supportive care is recommended and treatment with granulocyte-colony stimulating factor (G-CSF or filgastrim) should be considered [14]. The majority of patients respond within days of treatment [14], but neutropenia may recur on subsequent exposure. Following the public health advisories, fewer patients underwent invasive procedures such as bone marrow biopsies.

We also recommend that clinicians inquire about patients' recent cocaine use (see Appendix 2) and request levamisole testing if urine is obtained within 48 hours of last cocaine use. The diagnosis should still be considered when patients present with other coexisting health conditions (e.g. HIV).

Further research is needed to establish methods for cocaine users to detect the presence of levamisole and studies to quantify the levamisole dose required to produce neutropenia.

In conclusion, neutropenia associated with levamisole-tainted cocaine presents a significant, emerging public health problem in Canada. For clinicians, the awareness of the differential diagnosis for neutropenia can ensure timely diagnosis and appropriate management of cases.

## Competing interests

The authors declare that they have no competing interests.

## Authors' contributions

LK lead the public health investigation in Alberta, was primary author, developed the concept and design of study; collected, analyzed and interpreted the data; drafted and approved the final manuscript. JB lead the public health investigation in British Columbia, was secondary author, developed the concept and design of study; collected, analyzed and interpreted the data; and revised and approved the final manuscript. NS conducted the public health investigation in British Columbia, developed the concept and design of study; collected, analyzed and interpreted the data; and revised and approved the final manuscript. IA conducted the public health investigation in Alberta, collected, analyzed and interpreted the data; and revised and approved the final manuscript. DL discovered the association between cocaine, levamisole and neutropenia, collected, analyzed and interpreted the data; and revised and approved the final manuscript. SF conducted the public health investigation in Alberta, collected, analyzed and interpreted the data; and revised and approved the final manuscript. NZ discovered the association between cocaine, levamisole and neutropenia, and revised and approved the final manuscript. JT supervised the public health investigation in Alberta, developed the concept and design of study; analyzed and interpreted the data; and revised and approved the final manuscript.

## **Appendix 1**

### **Case Definitions**

• **Confirmed case**: laboratory confirmed exposure to cocaine and levamisole and neutropenia (neutrophil counts less than 0.5 per 10^9 ^cells/L).

• **Probable case**: laboratory confirmed or a history of exposure to cocaine and neutropenia; or levamisole positive and serious infection determined post-mortem.

• **Suspect case**: signs and symptoms common to neutropenia and a history of exposure to cocaine or levamisole.

## **Appendix 2**

### Enhanced interview questions related to **RECENT **cocaine use

• What type of cocaine (crack, powder) did you use?

• Did you smoke, snort, or inject?

• How long did you use (Number of days)?

• How often did you use (Number of times per day, week, month, year)?

• How much did you use (Number of grams/day)?

• Was there anything different in the look, taste, texture, smell, effect of the cocaine when last used?
